# Association between nontraditional lipid parameters and the risk of type 2 diabetes and prediabetes in patients with nonalcoholic fatty liver disease: from the national health and nutrition examination survey 2017–2020

**DOI:** 10.3389/fendo.2024.1460280

**Published:** 2024-08-30

**Authors:** Jierui Liu, Qingan Fu, Ruolin Su, Rixiang Liu, Shisheng Wu, Ke Li, Jianhua Wu, Nuobei Zhang

**Affiliations:** ^1^ Gastroenterology Department, The Second Affiliated Hospital of Nanchang University, Nanchang, Jiangxi, China; ^2^ Cardiovascular Medicine Department, The Second Affiliated Hospital, Jiangxi Medical College, Nanchang University, Nanchang, Jiangxi, China

**Keywords:** nonalcoholic fatty liver disease, lipid, type 2 diabetes mellitus, prediabetes, risk factors

## Abstract

**Background:**

Nonalcoholic fatty liver disease (NAFLD) is a prevalent metabolic disorder strongly linked to type 2 diabetes mellitus (T2DM). Understanding the predictive value of lipid parameters in identifying abnormal glucose metabolism in NAFLD patients is crucial for early intervention.

**Methods:**

This study analyzed data from the National Health and Nutrition Examination Survey(NHANES) database (2017-2020) involving 1066 NAFLD patients. Participants were categorized into three groups: T2DM (n=414), prediabetes mellitus (pre-DM) (n=507), and normoglycemia (NG) (n=145). Traditional lipid parameters [triglycerides (TG) and high-density lipoprotein cholesterol (HDL-C)] and nontraditional lipid parameters [atherogenic index of plasma (AIP), residual cholesterol (RC), and non-high-density lipoprotein cholesterol (non-HDL-C)] were evaluated for their association with T2DM and pre-DM.

**Results:**

Elevated TG levels were significantly associated with an increased risk of T2DM and pre-DM, whereas high HDL-C demonstrated a protective effect. Among nontraditional lipid parameters, increased AIP and RC were most strongly associated with T2DM risk, while high non-HDL-C was best associated with the development of pre-DM. Stratified analyses revealed that these associations were stronger in younger, non-obese, smoking, and female NAFLD patients.

**Conclusion:**

Nontraditional lipid parameters, particularly AIP and RC, show superior predictive value over traditional lipid parameters in identifying abnormal glucose metabolism in NAFLD patients. Incorporating these novel biomarkers into clinical practice could enhance early detection and prevention strategies for T2DM and pre-DM in this high-risk population.

## 1 Background

Nonalcoholic fatty liver disease (NAFLD) is a hepatic manifestation of metabolic syndrome that is pathologically characterized by an abnormally high accumulation of hepatic fat and excludes other etiological factors, such as alcohol consumption and viral hepatitis. During the last few decades, NAFLD has rapidly become the most prevalent liver disease, affecting approximately one-third of the world’s population, and is the main cause of high morbidity and mortality from liver-related diseases, causing severe health problems and financial burdens for patients (1). Type 2 diabetes mellitus (T2DM) is a metabolic disease characterized by hyperglycemia, hyperinsulinemia, and insulin resistance(IR) that affects hundreds of millions of individuals globally, and the association between NAFLD and T2DM has been well documented (2, 3). A meta-analysis of 501,022 individuals with a median follow-up time of 5 years suggested that NAFLD may lead to an approximately 2.2-fold increased risk of developing T2DM (4). A study by Kanwalet et al. revealed that NAFLD patients with T2DM have a greater than 2-fold higher risk of developing hepatocellular carcinoma or cirrhosis than nondiabetic patients (5). Before T2DM is clinically diagnosed, a large proportion of patients exhibit impaired fasting glucose or glucose tolerance, which is called prediabetes mellitus (pre-DM), and this condition is also considered to be strongly associated with NAFLD (6), which has potential for the progression to T2DM (7). Therefore, early glucose metabolism management in NAFLD patients is particularly important in clinical practice, yet there is no proper index that can effectively reveal glucose metabolism in NAFLD patients, and the research and development of a convenient predictor of abnormal glucose metabolism biometabolites has become imperative.

Abnormal lipid metabolism is the link between NAFLD and abnormal glycometabolism, and NAFLD is closely associated with lipid abnormalities (8), which are manifested by elevated triglyceride (TG) levels and low high-density lipoprotein cholesterol (HDL-C) levels. Moreover, total cholesterol (TC) metabolism is significantly altered in NAFLD patients, as reflected by both increased cholesterol synthesis and diminished absorption (9), and excessive cholesterol build-up leads to pancreatic β-cell dysfunction, disrupts glucose tolerance, and affects insulin secretion. In addition, hepatic fatty accumulation further exacerbates IR, forming a vicious cycle (10). In addition to the traditional lipids mentioned above, several studies have recently shown that nontraditional lipid parameters, such as the plasma atherogenic index (AIP), residual cholesterol (RC), and non-high-density cholesterol (non-HDL-C), are notably associated with NAFLD, T2DM, and pre-DM and, more importantly, have a better ability to predict the occurrence and progression of NAFLD and T2DM than traditional lipid parameters, which are expected to be reliable predictors of glucose metabolism abnormalities in patients with NAFLD (11–13). However, few studies have investigated the association between nontraditional lipid parameters, such as the AIP, and the risk of T2DM and pre-DM in the NAFLD population.

Based on a nationally representative sample-based database of the U.S. population, the aim of this study was to comprehensively analyze the association between nontraditional lipid parameters and the occurrence of T2DM and pre-DM in the NAFLD population to explore whether novel lipid parameters can be predictive factors of abnormal glucose metabolism in NAFLD patients and to help clinicians monitor and prevent the incidence of pre-DM and T2DM in the early stage among NAFLD patients.

## 2 Methods

### 2.1 Study population

The National Health and Nutrition Examination Survey (NHANES) is a major cross-sectional research project with multiple cycles of data in a two-year cycle led by the National Center for Health Statistics (NCHS), Centers for Disease Control and Prevention (CDC). Since 1999, the survey has been conducted annually on a sample of about 5,000 people in 15 different counties in the United States(US). The survey was approved by the Research Ethics Review Board of NCHS, and to ensure that the rights of participants were protected, NHANES has obtained informed written consent from all individuals participating in the study. We selected data from the NHANES database from 2017 through 2020, which contained 15,560 participants in this cycle (14). During the cycle, the NHANES staff used a FibroScan 502 Touch device to assess participants’ vibration-controlled transient elastography (VCTE) and measured ultrasound attenuation related to the extent of NAFLD and recorded the coefficient of attenuation parameter (CAP) as an indicator of the degree of hepatic steatosis.

### 2.2 Definitions of NAFLD, T2DM and pre-DM

In this study, we used the CAP threshold of 285 dB/m as an identification threshold for hepatic steatosis, which has been previously demonstrated to have 80% sensitivity and 77% specificity and is widely used for the detection of individual hepatic steatosis in the US population (15). We diagnosed NAFLD in patients with a CAP of more than 285 dB/m, excluding those with excessive alcohol consumption and other liver diseases.

According to the American Diabetes Association’s guidelines (16), the presence of any of the three following conditions indicates the presence of T2DM: (1) the use of oral antihyperglycemic medication or insulin; (2) a fasting blood glucose (FBG) greater than or equal to 126 mg/dL or a glycated hemoglobin (HbA1c) ≥ 6.5%; and (3) a self-reported history of diabetes mellitus. Pre-DM, on the other hand, was defined as an FBG value between 100-125 mg/dL or an HbA1c level of 5.7% - 6.4%.

**Figure 1:**
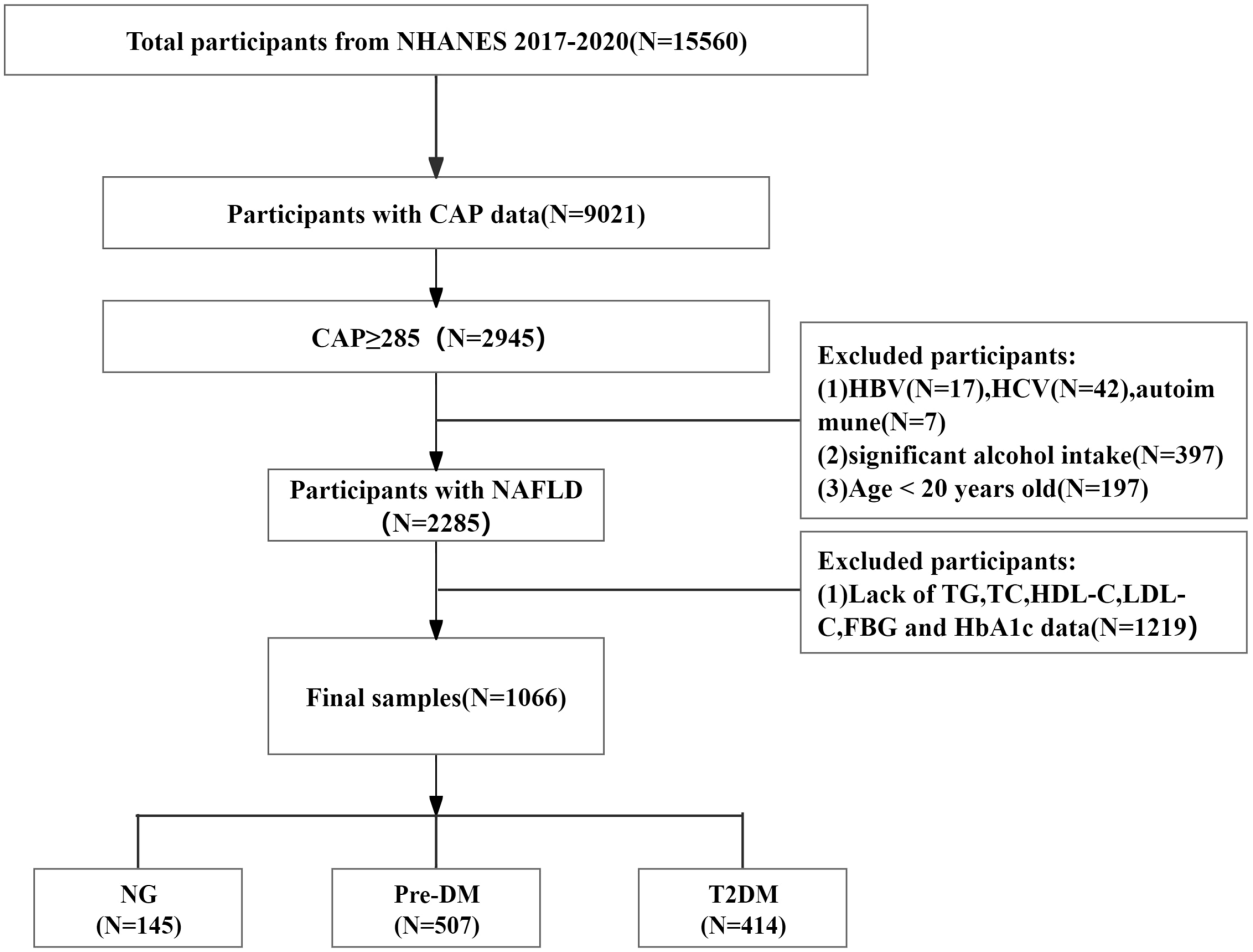
Flow chart of patient recruitment.

The exclusion criteria for participants were as follows: (1) did not have VCTE or failed VCTE results (n=6539); (2) had CAP <285 dB/m (n=6076); (3) tested positive for hepatitis B virus (HBV) surface antigen (n=17) or hepatitis C virus (HCV) Ribonucleic Acid(RNA)(n=42) or had autoimmune hepatitis (n=7); (4) consumed excessive alcohol (women > 10 g/day, men > 20 g/day, n=397); (5) were <20 years of age (n=197); and (6) had missing TG, TC, HDL-C, LDL-C, FBG, and HbA1c data (n=1219). Ultimately, a total of 1066 participants were included in the analysis, including 414 with T2DM, 507 with pre-DM, and 145 with normoglycemia (NG). A flowchart of the brief design of this study is presented in **Figure 1**.

### 2.3 Other covariates and definitions

Various demographic and health profile information was gathered from NHANES household interviews for participants in 2017–2020, including age, sex, race, educational level, smoking status, disease conditions, and substance use. Educational attainment was categorized into three classes: high school or less, college or associate degree, and college graduation or higher. Health technicians measured elevation, weight, and waist circumference and calculated body mass index (BMI) using a standardized protocol for participants at mobile examination centers (MECs). Blood pressure was measured three times at rest by trained physicians at MECs using a standardized protocol, and the mean systolic blood pressure(SBP) and diastolic blood pressure (DBP) were subsequently calculated.Laboratory tests were performed on various blood samples as detailed in the procedure manual [2017-March 2020 Pre-Pandemic Laboratory Data - Continuous NHANES (cdc.gov)]. HBA1C, FBG, HDL-C, TC, and TG levels were measured by the researchers. TC was measured enzymatically; serum TG and HDL-C were measured photometrically; and the researchers calculated LDL-C from direct measures of TC, TG, and HDL-C via the Friedewald equation and reported it as a separate variable in the dataset. In addition, covariates included albumin (ALB), alanine transferase (ALT), aspartate transaminase (AST), γ-glutamyltransferase (GGT), serum creatinine (Scr), blood urea nitrogen (BUN), and total bilirubin (TBIL).

A lifetime cumulative total of 100 or more cigarettes was defined as a smoker (17). The 2017 American Heart Association/American College of Cardiology (AHA/ACC) guidelines recommend that individuals with an SBP ≥130 mmHg and/or a DBP ≥80 mmHg be defined as hypertensive (18), and participants who were taking antihypertensive medication were also categorized as hypertensive. Participants were asked by trained interviewers, “Has a doctor or other health professional ever told you that you have coronary heart disease/angina/stroke/myocardial infarction?” If they answered “yes” to any of these questions, they were deemed to have Cardiovascular Disease (CVD) by NHANES researchers.

Nontraditional lipid parameters were calculated as follows:

Lipoprotein combined index (LCI)=TC×TG×LDL-C/HDL-C (19);

AIP=lg (TG/HDL-C) (20);

Non-HDL-C=TC−HDL-C (21);

Castelli’s index-I (CRI-I) =TC/HDL-C (22);

Castelli’s index-II (CRI-II) =LDL-C/HDL-C (22);

RC=TC−HDL-C−LDL-C (23).

### 2.4 Statistical analysis

Analysis of the data and visualization of the images were performed using R software (version 4.3.1). The normality of the data was examined using the Shapiro−Wilk test. Normally distributed continuous variables are represented as the mean ± standard deviation, and nonnormally distributed continuous variables are summarized as medians (quartiles). T tests or rank sum tests were used to compare differences between groups. Categorical variables are expressed as percentages, and the Pearson chi-square test was used for comparisons. A small number of missing values within 10% of the SBP and DBP data were filled in using multiple interpolation.

First, univariate logistic regression models were used to assess the effects of each variable on the risk of NAFLD combined with T2DM or pre-DM, and odds ratios (ORs) and corresponding 95% confidence intervals (CIs) were calculated. Next, two multifactorial logistic regression models were constructed for the T2DM and pre-DM groups. Model 1 was adjusted for baseline age and sex, and Model 2 was adjusted based on Model 1 with the addition of the adjusted covariates SBP, AST, history of hypertension, history of CVD, and history of smoking. In addition, a generalized additive model with fitted smoothness was used to characterize the dose−response relationships between lipid parameters and the risk of NAFLD in patients with T2DM and pre-DM. Subsequently, receiver operating characteristic (ROC) curves were constructed to estimate the predictive ability and accuracy of each lipid biomarker for the risk of combined T2DM or pre-DM in NAFLD patients and to determine the optimal cutoff value, we calculated the area under the ROC curve(AUC) and classification as poor(0.500-0.599), fair(0.600-0.699) and good(0.700-0.799).We performed stratified analysis based on Model 2 stratified according to sex (male, female), age (<60 years, ≥60 years), smoking status (yes, no), and BMI (<28, ≥28) and calculated AUC of each subgroup to identify the predictive value of lipid parameters for the risk of T2DM and pre-DM in various populations of NAFLD patients. A two-sided P<0.05 was considered to indicate statistical significance.

## 3 Results

### 3.1 Baseline characteristics of the study participants

Of the 1066 NAFLD participants, 507 (48%) were female, whereas 559 (52%) were male, with a median age of 55 years. **Table 1** shows the baseline characteristics of the participants after they were categorized in terms of their diabetes status. In comparison to those in the NG group, participants in the T2DM and pre-DM groups were more likely to be older, male, hypertensive, smokers, and CVD patients and had elevated levels of SBP, ALT, AST, BUN, GGT, TG, LCI, AIP, and RC, as well as decreased levels of HDL-C, compared to patients with normal blood glucose levels in the T2M and pre-DM groups. (all P<0.05).

**Table 1:** Baseline clinical characteristics according to diabetic status.

Characteristics	NG N=145	T2DM N=414	Pre-DM N=507	P-value
AGE(yr)	37.0(28.0-48.0)	62.0(53.0-69.0)	54.0(41.0-64.0)	<0.001
SEX(%)				0.005
Male	58(40.0)	223(53.9)	278(54.8)	
Female	87(60.0)	191(46.1)	229(45.2)	
RACE(%)				0.158
Mexican American	30(20.7)	77(18.6)	86(16.9)	
Other Hispanic	14(9.7)	48(11.6)	50(9.9)	
Non-Hispanic White	55(37.9)	129(31.2)	189(37.3)	
Non-Hispanic Black	20(13.8)	88(21.2)	97(19.1)	
Other Race	26(17.9)	72(17.4)	85(16.8)	
Education level(%)				0.080
High school or less	57(39.3)	202(48.8)	233(45.9)	
Some college or AA degree	57(39.3)	127(30.7)	156(30.8)	
College graduate or above	31(21.4)	85(20.5)	118(23.3)	
Smoking(%)				<0.001
Yes	48(33.1)	178(43.0)	218(43.0)	
No	97(66.9)	236(57.0)	289(57.0)	
Hypertension (%)				<0.001
Yes	67(46.2)	332(80.2)	328(64.7)	
No	78(53.8)	82(19.8)	179 (35.3)	
CVD(%)				<0.001
Yes	5(3.4)	84(20.3)	43(8.5)	
No	140(96.6)	330(79.7)	464(91.5)	
BMI(kg/m2)	32.8(29.0-38.4)	33.0(29.1-37.3)	32.0(28.2-36.9)	0.049
Waist(cm)	109.0 (98.9-118.6)	113.1(103.4-123.1)	108.1 (98.8-118.6)	<0.001
SBP(mmHg)	113.3(106.3-125.7)	126.7(116.1-142.3)	123.3 (114.8-134.7)	<0.001
DBP(mmHg)	75.3(70.0-82.3)	75.0(66.4-82.3)	76.0(69.7-84.0)	0.028
ALB(g/L)	40.0(38.0-42.0)	40.0(38.0-41.8)	40.0(38.0-42.0)	0.540
ALT(U/L)	18.0(13.0-28.0)	22.0(16.0-31.0)	21.0(16.0-32.0)	0.021
AST(U/L)	18.0(15.0-22.0)	19.0(15.0-25.0)	20.0(17.0-25.0)	0.004
BUN(mmol/L)	4.6(3.9-5.7)	5.4(4.3-7.1)	5.0(4.3-6.1)	<0.001
Scr(mmol/L)	70.7(60.1-81.3)	75.1(59.2-91.1)	75.1(61.9-86.6)	0.087
GGT(IU/L)	21.0(16.0-32.0)	27.0(20.0-43.0)	24.0(18.0-35.5)	<0.001
TBIL(mmol/L)	6.8(5.1-8.6)	6.8(5.1-10.3)	6.8(5.1-10.3)	0.012
FBG(mg/dl)	95.0(91.0-97.0)	143.0(126.0-175.8)	107.0(102.0-113.5)	<0.001
HbA1c (%)	5.3(5.2-5.5)	6.9(6.4-8.1)	5.7(5.5-5.9)	<0.001
HDL-C(mmol/L)	1.2(1.0-1.4)	1.1(1.0-1.3)	1.2(1.0-1.4)	0.006
TG(mmol/L)	1.1(0.8-1.6)	1.4(1.0-2.0)	1.3(0.9-1.8)	<0.001
LDL-C(mmol/L)	2.8(2.4-3.3)	2.4(1.9-3.2)	3.0(2.5-3.5)	<0.001
TC(mmol/L)	4.6 (4.2-5.1)	4.4(3.8-5.1)	5.0(4.3-5.6)	<0.001
LCl	11.7(6.6-21.6)	13.7(7.7-24.2)	16.3(8.9-27.2)	0.001
AIP	0(-0.2-0.1)	0.1(-0.1-0.3)	0(-0.1-0.2)	<0.001
Non-HDL-C	3.4(2.9-4.0)	3.2(2.6-3.9)	3.7(3.1-4.3)	<0.001
CRI	3.8(3.1-4.6)	3.8(3.1-4.7)	4.1(3.4-4.9)	0.002
CRII	2.3(1.8-3.0)	2.2(1.6-2.8)	2.5(1.9-3.2)	<0.001
RC	0.5(0.4-0.7)	0.6(0.5-0.9)	0.6(0.4-0.8)	<0.001

Data are presented as median (interquartile) or number (proportion, %).

NG, normoglycemic; Pre-DM, pre-diabetes; T2DM, type 2 diabetes; CVD, cardiovascular disease; BMI, body mass index; SBP, systolic blood pressure; DBP, diastolic blood pressure; ALB, albumin; ALT, alanine aminotransferase; AST, aspartate aminotransferase; BUN, blood urea nitrogen; Scr, serum creatinine; GGT, gamma-glutamyl transferase; TBIL, total bilirubin; FBG, fasting blood glucose; HbA1c, hemoglobin A1c;HDL-C, high-density lipoprotein cholesterol; TG, triglycerides; LDL-C, low-density lipoprotein cholesterol; TC, total cholesterol; LCI, lipoprotein combine index; AIP, atherogenic index of plasma; CRI-I, Castelli’s index-I;CRI-II, Castelli’s index-II; RC, remnant cholesterol.

### 3.2 Univariate associations between T2DM and pre-DM

Univariate analysis of outcomes indicated that age, sex, smoking status, hypertension status, CVD status, SBP, AST, TG, LDL-C, AIP, and RC were significantly associated with T2DM and pre-DM. Among traditional lipid parameters, TG was the most strongly associated risk factor in patients with NAFLD accompanied by T2DM and pre-DM (OR: 1.882; 95% CI 1.399-2.531) (OR: 1.456; 95% CI 1.116-1.899). However, for nontraditional lipid parameters, the AIP was the top risk factor related to T2DM (OR: 4.734; 95% CI 2.351-9.533), while the RC was the greatest risk factor linked to pre-DM (OR: 2.267; 95% CI 1.269-4.048) (**Table 2**).

**Table 2:** Associations between T2DM and pre-DM with univariate.

Variables	T2DM	95%CI	P-value	Pre-DM	95%CI	P-value
	OR			OR		
AGE	1.109	1.089-1.13	<0.001	1.056	1.042-1.071	<0.001
SEX						
Male	Reference			Reference		
Female	0.571	0.389-0.839	0.004	0.549	0.377-0.799	0.002
Smoking						
No	Reference			Reference		
Yes	1.524	1.025-2.267	0.037	1.524	1.034-2.247	0.033
Hypertension						
No	Reference			Reference		
Yes	4.714	3.140-7.075	<0.001	2.133	1.468-3.100	<0.001
Cardiovascular disease						
No	Reference			Reference		
Yes	7.127	2.830-17.949	<0.001	2.595	1.008-6.677	0.048
SBP	1.04	1.027-1.053	<0.001	1.036	1.022-1.05	<0.001
DBP	0.987	0.971-1.004	0.13	1.005	0.988-1.022	0.567
BMI	1.003	0.977-1.029	0.846	0.984	0.96-1.009	0.218
WAIST	1.019	1.006-1.032	0.005	1	0.987-1.013	0.994
ALB	0.988	0.932-1.048	0.688	1.005	0.946-1.069	0.863
ALT	1.013	1.0-1.027	0.051	1.014	1.001-1.027	0.03
AST	1.023	1.001-1.046	0.043	1.034	1.009-1.059	0.008
BUN	1.247	1.117-1.393	<0.001	1.054	0.942-1.179	0.358
Scr	0.999	0.996-1.002	0.6	0.998	0.995-1.001	0.201
GGT	1.009	1.001-1.017	0.021	1.004	0.997-1.01	0.27
TBIL	1.063	1.014-1.115	0.011	1.043	0.994-1.094	0.088
HDL-C	0.522	0.282-0.968	0.039	0.872	0.473-1.606	0.66
TG	1.882	1.399-2.531	<0.001	1.456	1.116-1.899	0.006
LDL-C	0.784	0.648-0.95	0.013	1.347	1.068-1.699	0.012
TC	0.88	0.739-1.047	0.149	1.392	1.132-1.712	0.002
LCI	1.011	0.999-1.022	0.077	1.02	1.007-1.033	0.002
AIP	4.734	2.351-9.533	<0.001	2.228	1.156-4.294	0.017
Non-HDL-C	0.924	0.774-1.103	0.382	1.416	1.151-1.742	0.001
CRI	1.058	0.897-1.248	0.5	1.268	1.067-1.506	0.007
CRII	0.897	0.737-1.091	0.277	1.284	1.034-1.595	0.024
RC	3.971	2.079-7.585	<0.001	2.267	1.269-4.048	0.006

OR, odds ratios; CI, confidence interval.

### 3.3 Associations between T2DM and pre-DM and lipid parameters

**Figure 2:**
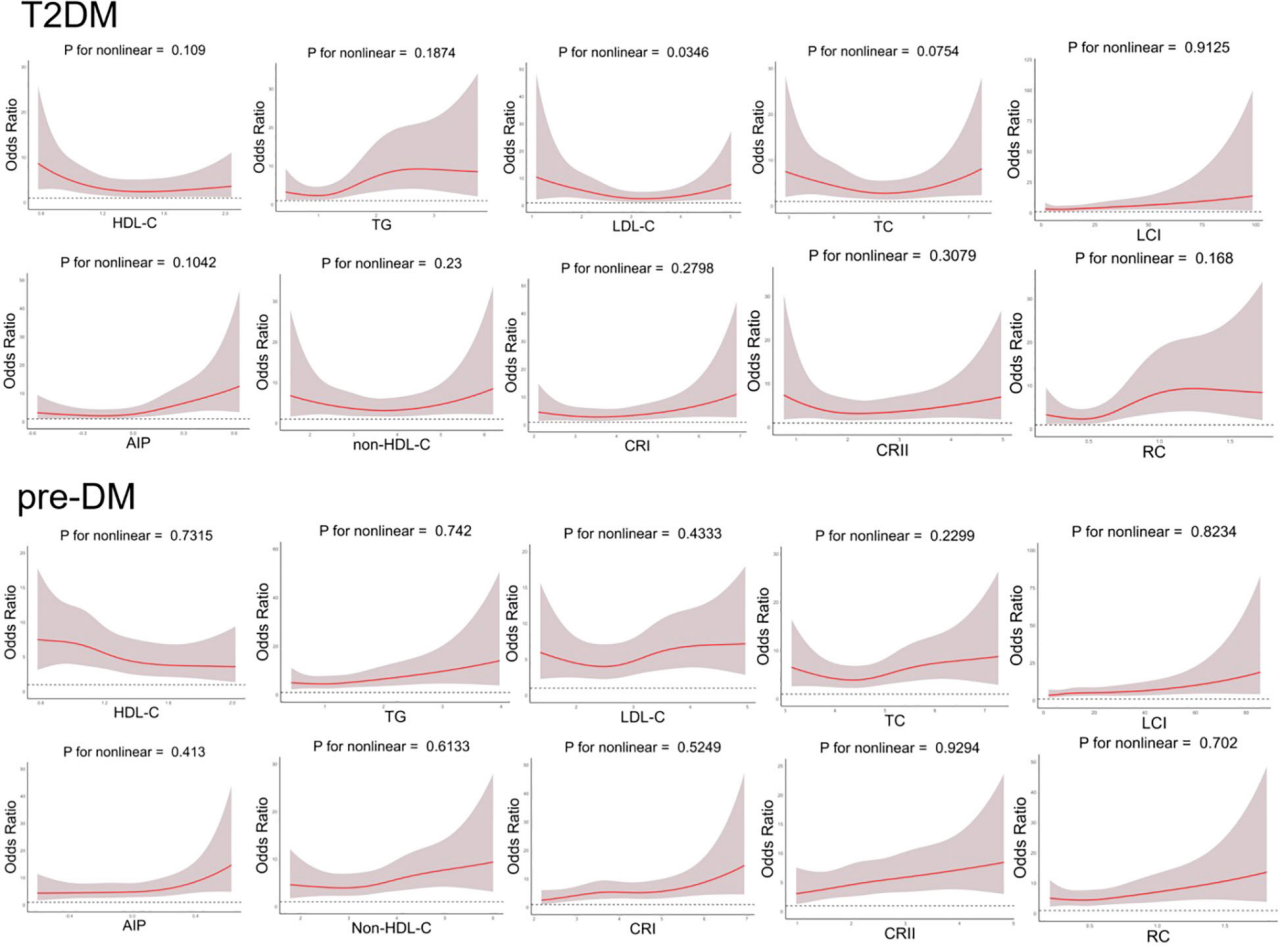
Generalized additive model with fitting smoothness for the dose-response relationship between lipid parameters and T2DM and pre-DM risk.

To account for possible bias brought about by the interaction between variables, we also performed multivariate logistic regression modeling to further assess the relationship between lipid parameters and the risk of T2DM and pre-DM (**Tables 3**, **4**). After adjusting for age, sex, smoking status, hypertension status, SBP, CVD status, and AST status, Model 2 revealed that among the traditional lipid parameters, increased HDL-C may be a protective factor (OR: 0.204, 95% CI 0.085-0.488) against T2DM and pre-DM (OR: 0.465, 95% CI 0.218-0.992); conversely, TG elevation (OR: 1.964; 95% CI 1.359-2.837) (OR: 1.367; 95% CI 1.034-1.808) was positively associated with T2DM and pre-DM risk. All of the nontraditional lipid parameters were positively associated with T2DM and pre-DM risk (except non-HDL-C with T2DM). Most importantly, the AIP showed the greatest risk factor (OR: 6.983, 95% CI 2.739-17.802) related to T2DM and pre-DM risk (OR: 2.278, 95% CI 1.089-4.765), followed by RC (OR: 4.353, 95% CI 1.948-9.728) (OR: 1.976, 95% CI 1.074-3.634), which were more strongly associated with T2DM than with traditional lipid parameters. **Figure 2** shows the linear relationship between lipid parameters and the risk of T2DM and pre-DM. Except for LDL-C, which demonstrated a significant nonlinear relationship with T2DM risk after smooth spline fitting (P for nonlinearity <0.05), no significant nonlinear relationships were observed between any of the lipid parameters and T2DM or pre-DM risk.

**Table 3:** Multivariate logistic regression analyses for the associations between lipid parameters with T2DM.

	Model 1	P value	Model 2	P value
	OR (95% CI)		OR (95% CI)	
HDL-C	0.179(0.076-0.422)	<0.001	0.204(0.085-0.488)	<0.001
TG	2(1.405-2.847)	<0.001	1.964(1.359-2.837)	<0.001
LDL-C	0.976(0.750-1.270)	0.856	0.963(0.732-1.268)	0.789
TC	1.012(0.799-1.282)	0.921	1.004(0.784-1.285)	0.976
LCI	1.025(1.009-1.041)	0.002	1.025(1.008-1.041)	0.004
AIP	7.611(3.076-18.835)	<0.001	6.983(2.739-17.802)	<0.001
Non-HDL-C	1.167(0.913-1.491)	0.217	1.15(0.890-1.487)	0.285
CRI	1.497(1.191-1.883)	0.001	1.481(1.164-1.883)	0.001
CRII	1.371(1.042-1.803)	0.024	1.338(1.002-1.787)	0.049
RC	4.532(2.096-9.801)	<0.001	4.353(1.948-9.728)	<0.001

Model 1: adjusted for age and sex at baseline; Model 2: further adjusted for age, sex, smoking status, hypertension status, CVD status, SBP and AST.

**Table 4:** Multivariate logistic regression analyses for the associations between lipid parameters with pre-DM.

	Model 1	P value	Model 2	P value
	OR (95% CI)		OR (95% CI)	
HDL-C	0.458(0.220-0.954)	0.037	0.465(0.218-0.992)	0.048
TG	1.46(1.110-1.921)	0.007	1.367(1.034-1.808)	0.028
LDL-C	1.222(0.949-1.575)	0.12	1.183(0.912-1.534)	0.206
TC	1.241(0.991-1.555)	0.06	1.189(0.942-1.500)	0.145
LCI	1.02(1.007-1.034)	0.003	1.017(1.003-1.030)	0.015
AIP	2.678(1.305-5.495)	0.007	2.278(1.089-4.765)	0.029
Non-HDL-C	1.333(1.062-1.675)	0.013	1.271(1.006-1.605)	0.045
CRI	1.353(1.113-1.645)	0.002	1.292(1.058-1.576)	0.012
CRII	1.353(1.058-1.730)	0.016	1.29(1.004-1.658)	0.046
RC	2.283(1.255-4.156)	0.007	1.976(1.074-3.634)	0.028

Model 1, adjusted for age and sex at baseline; Model 2, further adjusted for age, sex, smoking status, hypertension status, CVD status, SBP and AST.

### 3.4 Performance of lipid parameters in predicting T2DM and pre-DM in NAFLD patients

**Figure 3:**
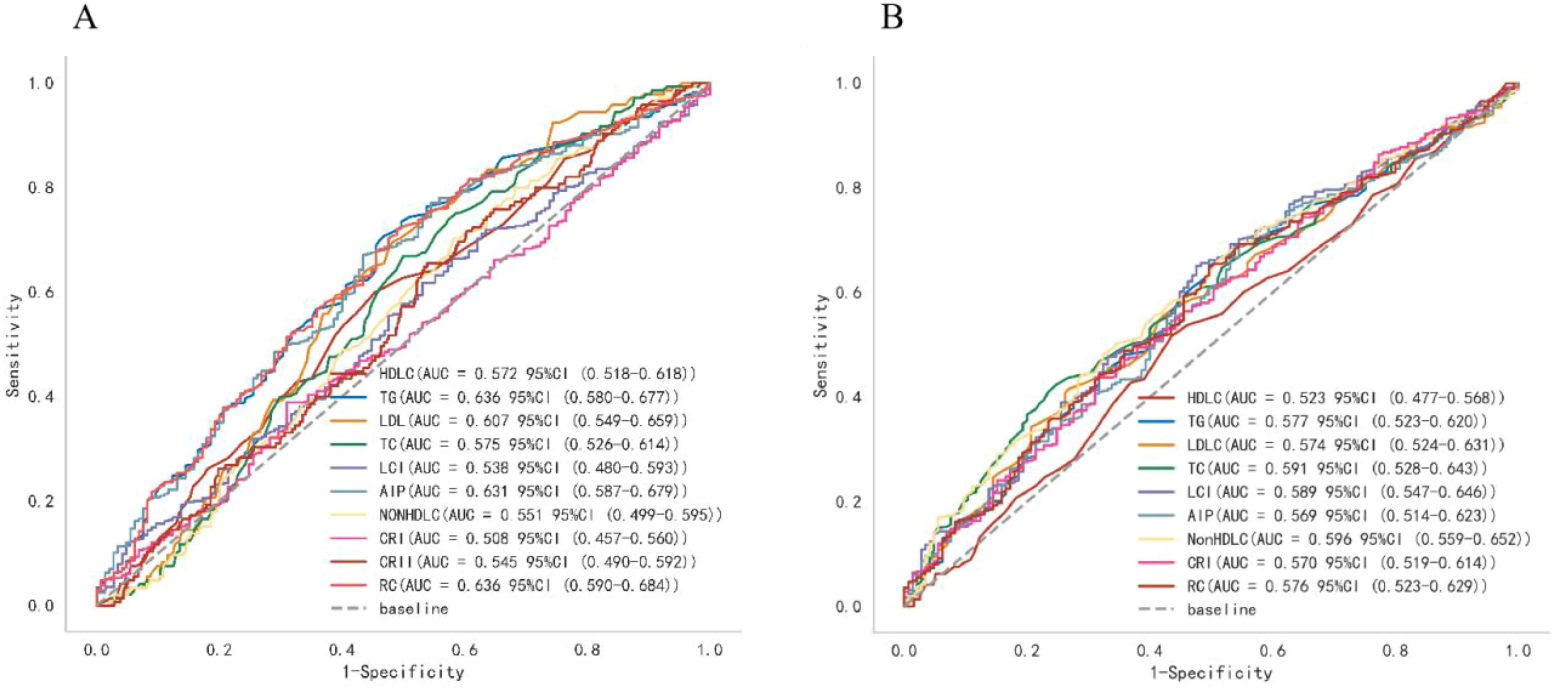
ROC curve analysis of the lipid parameters in predicting T2DM **(A)** and pre-DM **(B)**.

Analysis of ROC curves was utilized to compare the accuracy of various lipid biomarkers in predicting the occurrence of T2DM and pre-DM (**Figure 3**; **Supplementary Table 1**; **Table 2**). The AUC of all lipid parameters for either T2DM or pre-DM exceeded 0.5, indicating their potential value in T2DM and pre-DM risk prediction. According to our classification of AUC, TG, AIP and RC were fair predictors of T2DM,better than other lipid parameters. While in pre-DM, non-HDL-C showed better predictive value than all other lipid parameters. Notably, among the nontraditional lipid parameters, RC showed the optimal ability to recognize T2DM with an AUC of 0.636 (0.583-0.686), an optimal threshold of 0.512, a specificity of 0.510, and a sensitivity of 0.720, whereas non-HDL-C outperformed all of the traditional lipid parameters in the recognition of pre-DM with an AUC of 0.596 (0.547-0.647), an optimal threshold of 3.750, a specificity of 0.676 and a sensitivity of 0.483.

### 3.5 Stratified analyses

**Figure 4:**
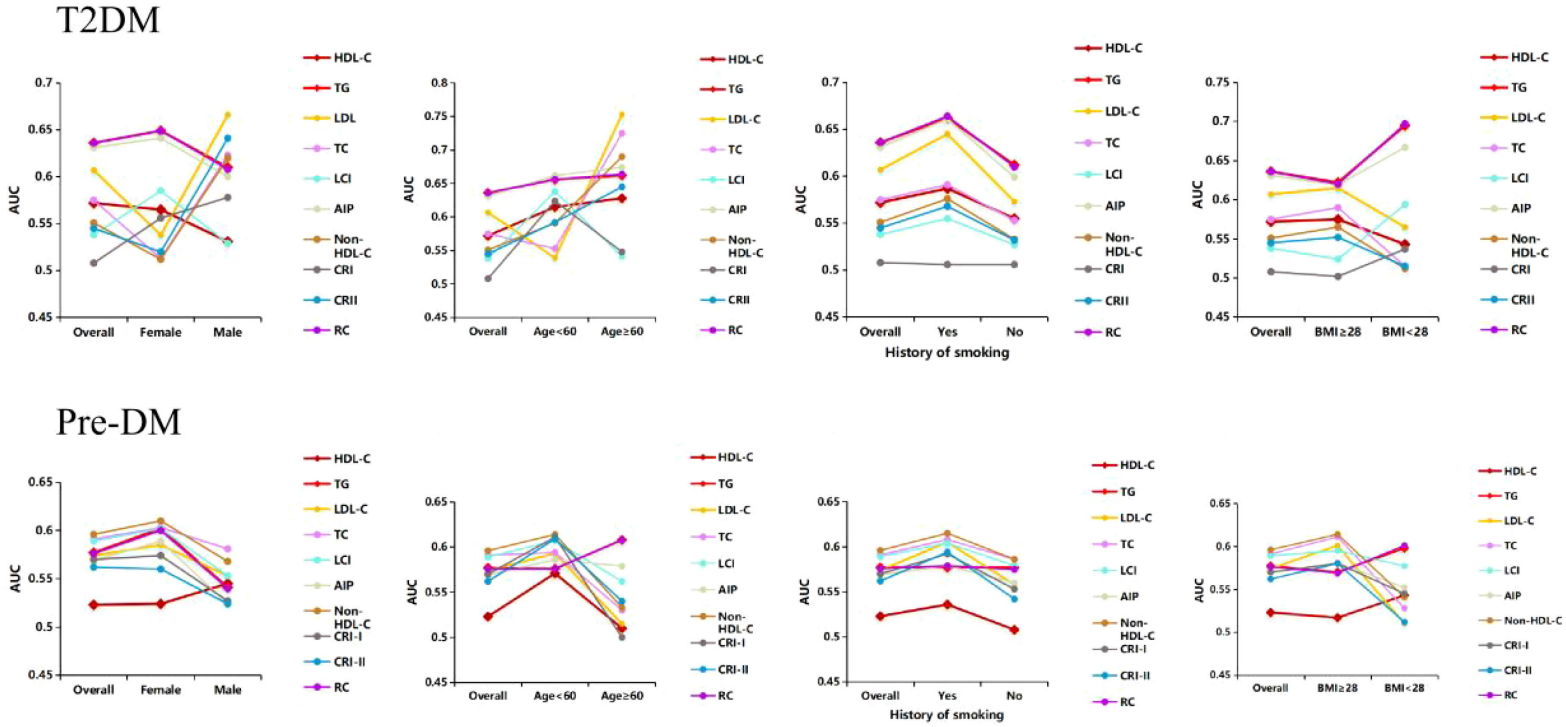
The AUC of lipid parameters in stratified analysis by sex, age, history of smoking and BMI.

Stratified analysis was conducted according to sex, age, smoking status, and BMI to assess the ability of lipid parameters to identify distinct populations (**Figure 4**). In Pre-DM, non-HDL-C appeared to show a reliable predictive value. A stronger association between most lipid parameters and the risk of pre-DM was observed in those aged <60 years, females, smokers, and those with a BMI ≥28. In contrast, in patients with T2DM, RC showed better predictive efficacy than other lipid parameters. Notably, in the smoking population, all lipid markers showed a stronger association with T2DM risk. Overall, nontraditional lipid parameters displayed better predictive efficacy than traditional lipid parameters in all subgroups.

## 4 Discussion

To the best of our knowledge, this is the first study that comprehensively describes the association of traditional and nontraditional lipid parameters with glucose metabolism levels in patients with NAFLD. The results revealed that after adjusting for confounders, TG, a traditional lipid parameter, was an independent risk factor for the development of T2DM and pre-DM in NAFLD patients, whereas HDL-C had a protective effect. On the other hand, all nontraditional lipid parameters were independent risk factors for T2DM and pre-DM in NAFLD patients, except for non-HDL-C, which was not associated with the risk of T2DM. Overall, nontraditional lipid parameters demonstrated better predictive value for the risk of T2DM and pre-DM in NAFLD patients compared to traditional lipid parameters.

Lipid metabolism disorders are often comorbid in patients with NAFLD and are mainly characterized by elevated TG and LDL-C levels, reduced HDL-C concentrations (24, 25), and overaccumulation of TC (9). Previous findings have suggested that lipotoxicity, inflammation and mitochondrial oxidative stress can induce IR (26–28). T2DM is a chronic metabolic disease characterized by insufficient insulin secretion due to IR or other causes. Pre-DM is an intermediate stage between T2DM and NG, which, if not treated promptly, will progress to T2DM at a rate of 5% to 10% per year (29). A considerable number of previous studies have explored the relationship between traditional lipid parameters and diabetes and concluded that high levels of TG and low levels of HDL-C can significantly increase the risk of diabetes (30–32), and the value of TG/HDL-C as a derivative of these two parameters has also been widely reported. A large multicenter retrospective cohort study in China noted that elevated TG/HDL-C is strongly associated with T2DM and pre-DM in patients with coronary artery disease, and the associations persisted after adjusting for variables such as sex, age, and smoking status (33). The AIP is defined as the logarithm of the ratio of TG to HDL-C, and another nationally representative cross-sectional study has shown a positive nonlinear association between the AIP and the risk of T2DM (34). Wang et al. reported that non-HDL-C was significantly and positively associated with the risk of T2DM in a rural H-type hypertensive population in Northeast China (35). Other researchers have observed that RC is positively associated with the risk of T2DM even when TC, LDL-C and HDL-C levels are normal (36). However, there is no comprehensive comparison or evaluation of the value of nontraditional versus traditional lipid parameters in identifying T2DM and pre-DM in the NAFLD population. The present study fills this lacuna and provides strong evidence that the predictive value of nontraditional lipid metrics such as the AIP, RC, and non-HDL-C is superior to that of traditional lipid parameters.

The mechanism by which TG is intrinsically linked to NAFLD combined with T2DM or pre-DM is related to IR. In NAFLD, abnormalities in lipid metabolism, such as increased TG production and excessive uptake of hepatic free fatty acids(FFAs), cause the accumulation of intrahepatic fat (37), which leads to inflammation, the induction of oxidative stress and the production of abnormal lipid molecules affecting signaling pathways, such as leptin, lipocalin and retinol-binding protein-4, eventually leading to IR (38, 39). In parallel, the buildup of TG leads to an increase in FFAs, causing alterations in pancreatic α-cell insulin signaling and overproduction of glucagon, consequently resulting in IR (40). Conversely, IR inhibits TG catabolism, which in turn can increase the level of FFAs in the liver, further exacerbating IR. The vicious cycle between TG levels and IR in NAFLD patients promotes reduced glucose tolerance and ultimately the development of pre-DM and T2DM. Alternatively, HDL-C and Apo A-I (the main protein component of HDL-C) inhibit cytokine- or glucose-induced apoptosis in β-cells and promote insulin secretion by means of the S1P signaling pathway (41), and the reduction in HDL-C in patients with NAFLD further promotes glucose metabolism disorders. RC is the TC that remains in the bloodstream after the body has accomplished a wide variety of physiological processes. Excess RC in plasma can penetrate the arterial wall, be absorbed by macrophages and smooth muscle cells, form foam cells and cause inflammation after stimulation by lipoprotein lipase, consequently elevating blood glucose (42).

Furthermore, in stratified analyses, we detected that most of the lipid parameters tended to be more reliable in predicting the risk of pre-DM in young, nonobese, smoking and female NAFLD patients, a finding that is in line with previous studies. Compared to elderly individuals, younger individuals typically have a high-animal diet and a sedentary lifestyle, which is an important contributor to IR (43, 44). Previous studies have revealed that smoking is positively associated with TG and LDL-C and negatively associated with HDL-C and HDL particle size (45). A meta-analysis involving 5898795 participants showed that an estimated 11.7% of male cases of T2DM and 2.4% of female cases of T2DM were attributable to smoking (46). Jeremy et al. reported that nonobese patients with NAFLD had severe dyslipidemia or impaired glucose tolerance, even more so than obese patients with NAFLD (47, 48). In this study, 75% of women diagnosed with pre-DM were over 45 years old, indicating that postmenopausal women are more susceptible to impaired glucose tolerance. This is probably due to the decrease in estrogen levels and the increase in androgen ratios in women after menopause, and this hormone disruption promotes central fat deposition (49), which exacerbates visceral obesity and IR and markedly increases the risk of impaired glucose tolerance in postmenopausal women (50).

Several limitations of this paper are not negligible. First, as a cross-sectional retrospective study, the impact of confounding factors could not be completely circumvented, and a causal relationship between lipid parameters and the risk of T2DM and pre-DM in patients with NAFLD has not yet been inferred. Moreover, the NHANES database, as the data source for this research, is primarily a population in the Americas, so further research is necessary to confirm the generalizability of the findings to other populations. Finally, there is a shortage of long-term follow-up data on lipid variables to explore the association between lipid parameters and the risk of T2DM and pre-DM in NAFLD patients over time, and larger multicenter clinical studies with longer follow-up durations are still needed to validate our findings in the future.

## 5 Conclusion

The study demonstrated that nontraditional lipid parameters, particularly AIP, non-HDL -C(only with pre-DM), and RC, have stronger associations with the risk of abnormal glucose metabolism in patients with NAFLD than traditional lipid parameters, and are expected to be predictors of the development of T2DM or pre-DM in patients with NAFLD in the future. Clinicians could consider the use of these novel biomarkers to better monitor and manage glucose metabolism in this high-risk population, potentially reducing the burden of diabetes-related complications.

## Data Availability

The original contributions presented in the study are included in the article/**Supplementary Material**. Further inquiries can be directed to the corresponding author.
