# Supplementary material for: Association between nontraditional lipid parameters and the risk of type 2 diabetes and prediabetes in patients with nonalcoholic fatty liver disease: from the national health and nutrition examination survey 2017–2020
Source: Front Endocrinol (Lausanne). 2024 Aug 30;15:1460280. doi: 10.3389/fendo.2024.1460280 (PMC11392789; doi:10.3389/fendo.2024.1460280)
Supplement: Supplementary file 1 [file Table1.docx]

| **Supplementary Table 1.**The AUC, best threshold, sensitivity, and specificity of lipid parameters in identifying T2DM | | | | | |
| --- | --- | --- | --- | --- | --- |
| **Variables** | **AUC** | **Sensitivity** | **Specificity** | **Best threshold** | **classification** |
| HDL-C | 0.572 | 0.6 | 0.546 | 1.16 | Poor |
| TG | 0.636 | 0.708 | 0.531 | 1.129 | Fair |
| LDL-C | 0.607 | 0.697 | 0.517 | 2.483 | Fair |
| TC | 0.575 | 0.669 | 0.5 | 4.37 | Poor |
| LCI | 0.538 | 0.618 | 0.469 | 10.842 | Poor |
| AIP | 0.631 | 0.671 | 0.566 | -0.003 | Fair |
| Non-HDL-C | 0.551 | 0.703 | 0.418 | 3.03 | Poor |
| CRI | 0.508 | 0.389 | 0.69 | 4.261 | Poor |
| CRII | 0.545 | 0.655 | 0.461 | 2.095 | Poor |
| RC | 0.636 | 0.72 | 0.51 | 0.512 | Fair |
| AUC:area under the receiver operating characteristic curve | | | | |  |
| **Supplementary Table 2.**The AUC, best threshold, sensitivity, and specificity of lipid parameters in identifying pre-DM | | | | | |
| **Variables** | **AUC** | **Sensitivity** | **Specificity** | **Best threshold** | **classification** |
| HDL-C | 0.523 | 0.517 | 0.564 | 1.24 | Poor |
| TG | 0.577 | 0.651 | 0.503 | 1.095 | Poor |
| LDL-C | 0.574 | 0.416 | 0.731 | 3.233 | Poor |
| TC | 0.591 | 0.424 | 0.752 | 5.15 | Poor |
| LCI | 0.589 | 0.651 | 0.524 | 12.109 | Poor |
| AIP | 0.569 | 0.704 | 0.441 | -0.097 | Poor |
| Non-HDL-C | 0.596 | 0.483 | 0.676 | 3.75 | Poor |
| CRI | 0.57 | 0.438 | 0.69 | 4.235 | Poor |
| CRII | 0.562 | 0.592 | 0.545 | 2.345 | Poor |
| RC | 0.576 | 0.655 | 0.497 | 0.495 | Poor |
| AUC:area under the receiver operating characteristic curve | | | | |  |
